# A Novel UHPLC-MS/MS Method for Measuring 8-*iso*-Prostaglandin F_2α_ in Bronchoalveolar Lavage Fluid

**DOI:** 10.3389/fchem.2021.695940

**Published:** 2021-08-12

**Authors:** Cory Holder, Aaron Adams, Claire Allison, Olivia Cote, Rachel Lippens, Benjamin C Blount, Lanqing Wang

**Affiliations:** ^1^ Tobacco and Volatiles Branch, Division of Laboratory Sciences, National Center for Environmental Health, Centers for Disease Control and Prevention, Atlanta, GA, United States; ^2^ Oak Ridge Institute for Science and Education, Oak Ridge, TN, United States

**Keywords:** EVALI (e-cigarette or vaping product use-associated lung injury), 8-isoprostane, UHPLC-MS/MS, BAL fluid, oxidative stress

## Abstract

In August 2019, the Centers for Disease Control and Prevention (CDC) received the first reports of lung injuries that were eventually termed e-cigarette, or vaping, product use–associated lung injury (EVALI). As part of the investigation, CDC laboratories rapidly developed assays for analyzing substances in bronchoalveolar lavage (BAL) fluid collected from EVALI case patients. This report describes the development and validation of a high-throughput isotope dilution UHPLC-MS/MS method for measuring a major oxidative stress biomarker, 8-iso-prostaglandin F_2α_ (8-isoprostane), in BAL fluid samples. The method showed good sensitivity, 17.6 pg/ml LOD, and requires only 50 μl of sample volume. The method had high throughput with an analytical run time of 11 min. The within-day and between-day coefficient of variation (CV) were below 2%. Accuracy, calculated from spiked recovery, at three spiking levels, ranged from 95.5–101.8%. This novel UHPLC-MS/MS method characterizes oxidative stress in lung epithelial tissue and thus helps to elucidate potential pathologic processes.

## Introduction

The Centers for Disease Control and Prevention (CDC) received initial case reports of a range of pulmonary illnesses requiring hospitalization in otherwise healthy users of e-cigarette, or vaping, products (EVPs) in August 2019. The number of hospitalized cases eventually rose to 2,807 with 68 confirmed deaths. (C (2020). Outbreak of L, 2020) The disease was initially attributed to an “unknown chemical exposure” and was eventually found to be strongly linked to inhaled Vitamin E acetate in vaping products. (Schier et al., 2019) EVALI patients were generally healthy before onset of symptoms, which included inflammation. (Krishnasamy et al., 2020) Inflammation can lead to oxidative stress (OS) which could contribute to the acute lung injury observed in EVALI case patients. (Imai et al., 2008) OS is characterized as an imbalance between reactive oxygen species (ROS) and anti-oxidants. ROS are unstable chemical compounds generated endogenously by immune responses, mitochondrial metabolism, and exposure to environmental toxicants such as tobacco smoke. (Ray et al., 2012) Elevated 8-*iso*-prostaglandin F_2α_ (8-isoprostane) concentrations are indicative of OS and have been linked to the pathophysiology of many diseases including neurodegenerative diseases, lung diseases, and cancers. (Montuschi et al., 1998)^,^ (Malli et al., 2013)^,^ (Cracowski et al., 2002)^,^ (Baraldi et al., 2003)^,^ (Montuschi et al., 2000) To analyze 8-isoprostane, bronchoalveolar lavage (BAL) fluid was obtained via bronchoscopy by spraying normal saline onto the lung epithelial lining and then applying mild suction to retrieve a fraction of that saline along with components from the lung epithelial lining fluid. This report highlights the rapid development and validation of a novel isotope dilution UHPLC-MS/MS method measuring 8-isoprostane in BAL fluid. Application of this method allows for a useful measure of OS in the lung epithelial lining and thus provides insights about the pathophysiology of EVALI. (Blount et al., 2020).

## Materials and Methods

### Chemicals

Acetonitrile (HPLC grade), methanol (HPLC grade), formic acid (≥ 99.5%), ammonium hydroxide (certified ACS plus), and phosphate-buffered saline (PBS) without calcium and magnesium were purchased from Fisher Scientific (NJ, United States). Water (HPLC grade) was purchased from JT Baker (NJ, United States). Synthetic 8-*Iso*-prostaglandin F_2α_ (8-isoprostane; CAS# 27415–26–5), 8-*iso*-prostaglandin F_2α_-*d*_4_ (>99%) (8-isoprostane d_*4*_; CAS# 211105–40–7), ent-8-iso-15(S)-PGF_2α_ (≥98%) (CAS# 214748–66–0); ent-8-iso-PGF_2α_ (≥98%) (CAS# 159812–83–6), 8-iso-PGE_1_ (≥98%) (CAS# 21003–46–3); PGE_1_ (≥98%) (CAS# 745–65–3); 8-iso-15(R)-PGF_2α_ (≥98%) (CAS# 214748–65–9); PGF_2α_ (≥98%) (CAS# 551–11–1); and 15(R)-PGF_2α_ (≥98%) (CAS# 37658–84–7) were obtained from Cayman Chemical Company (MI, United States). Potassium phosphate monobasic crystals (Reagent ACS) was obtained from Acros (NJ, United States). Potassium phosphate buffer with a pH of 6.1 was prepared using a Mettler Toledo S220 pH meter (Greifensee, Switzerland).

### Standard Solutions

The initial 8-isoprostane stock solution was prepared by adding 7.05 mg of the dry powder (99% purity) into a 200 ml volumetric flask and diluting with methanol in HPLC water (v/v 1:1) to obtain a native spiking solution concentration of 34.9 μg/ml. Working solutions were prepared for standards from serial dilutions of initial native and ISTD stock solutions with methanol and HPLC water (v/v 1:1). Standards were prepared at 10 concentrations ranging from 0 to 1,410 pg/ml by serial dilution of working solutions with methanol and HPLC water (v/v 1:9) and stored in 1.5 ml amber glass vials at −70°C. The materials for the standard solutions were all prepared gravimetrically, and mass results were reported on the conventional basis for weighing in air. These standard solutions were only used as external calibration standards for each analytical run.

### Internal Standard Solutions

The isotopically labeled internal standard (ISTD), 8-isoprostane *d*_*4*_, was dissolved in methyl acetate (100 μg/ml), then 0.5 ml of the initial solution was added to a 100-ml volumetric flask and diluted with methanol in HPLC water (v/v 1:1) resulting in a working solution with a concentration of 500 ng/ml. The final ISTD solution was made by adding 60 ml of the ISTD working solution to a 2,000 ml volumetric flask and diluting with methanol in HPLC water (v/v 1:9), bringing the final concentration to 15 ng/ml. The ISTD solution was dispensed into 2 ml cryovials, stored at −70°C, and thawed before sample preparation.

### Anonymous Bronchoalveolar Lavage Fluid Collection

Non-EVALI BAL fluids used in this study were acquired from Discovery Life Sciences (Huntsville, AL, United States). They were shipped frozen on dry ice and then stored in −70°C freezers until analyzed.

### Sample Stability

Individual BAL fluids were screened and three BAL fluid samples with no detectable 8-isoprostane levels were selected to make a blank pool for accuracy, precision, and stability testing. Native 8-isoprostane was dissolved into methanol and water (v/v 1:9) to make spiking solutions. These solutions were spiked into the pooled BAL fluid to achieve six final pools with concentrations ranging from 0–2,000 pg/ml. The spiked pools were used for all method validation experiments. One of the BAL fluid samples with no detectable 8-isoprostane was spiked to create a series of four individual BAL fluid spiked levels with concentrations from 0–2,000 pg/ml. This spiked individual sample was used for LOD testing.

### Saline Quality Controls

Because the available quantity of BAL fluid was not sufficient to perform all experiments, blanks and QC pools were prepared using phosphate-buffered saline (PBS). The PBS solution was spiked with native 8-isoprostane to form low, medium, and high QC concentrations of 200, 500, and 2,000 pg/ml, respectively. The saline QCs were processed in every analytical run and monitored for accuracy and precision.

### Sample Preparation

An analytical run consisted of a blank, three quality controls (low, medium, and high), and up to 44 unknown BAL fluid samples, and each 96-well plate could hold two analytical runs. Ten external calibration standards were run in duplicate for each analytical run.

BAL fluid was thawed and centrifuged at 1,500 rpm for 12 min at 4°C, and the supernatant was transferred to 2 ml Nalgene cryovials. Prior to aliquoting, each BAL fluid sample was vortexed for approximately 10 s to homogenize the sample. A Hamilton Starlet system was utilized for the automated liquid transfer of internal standard, phosphate buffer, water, and methanol. Liquid transfers were performed using 50, 300, and 1,000 µl black conductive pipette tips from Hamilton in which 40 μl of the isotopically labeled internal standard working solution (15 ng/ml), 160 μl of buffer solution (0.5 M phosphate buffer, pH 6.1), and 1,150 μl of HPLC water, respectively, were dispensed into glass test tubes (12 × 75 mm). Due to variations in sample consistency, a manual transfer of 50 μl of BAL fluid was performed using 250 μl Ranin Precision Tips. A sample volume of 50 μl represents a 20 fold dilution and an appropriate correction factor was applied to the measured concentration. Finally, 400 µl of methanol was added to each sample tube. The entire contents in the glass tube were transferred to a 96-well weak anion exchange SPE plate using the Hamilton Starlet system. SPE cleanup was done using the Strata-X-AW 33 µm Polymeric Weak Anion, 60 mg/ 96-well plate from Phenomenex (Torrance, CA, United States). A Biotage Pressure + 96 positive pressure manifold (Biotage, Charlotte, NC, United States) using nitrogen gas generated in-house with a NM20ZA Peak Generator was used to apply positive pressure to the SPE plate. The SPE plate was washed with 1.8 ml of HPLC water, followed by a 1.8 ml solution of methanol in HPLC water (v/v 1:3), and finally 1.8 ml of acetonitrile. Samples were then eluted using methanol and collected in an Advantage Series SiliGuard coated 2 ml 96 deep square well collection plate with a tapered V-bottom (Analytical Sales and Services Inc., Flanders, NJ, United States), evaporated under nitrogen flow at 37°C, reconstituted with 50 μl of 25% methanol in water, vortexed lightly for approximately 2 min, and subsequently injected into the LC-MS/MS.

### UHPLC-MS/MS

The LC-MS/MS instrument parameters were kept the same as our previously published CLIA urinary assay. (Holder et al., 2020) In brief, chromatographic separation was achieved using a Waters ACQUITY reversed-phase column (150 mm × 2.1 mm, particle size 1.8 μm, C18) and a Waters ACQUITY reversed-phase pre-column (5 mm × 1 mm, particle size 1.7 μm, C18) (Milford, MA, United States) on an ultra high performance liquid chromatographic system from Shimadzu Corp. (Columbia, MD, United States) A gradient program was performed with a combination of 0.15% formic acid in water (mobile phase A) and acetonitrile in 0.15% formic acid in water (v/v 1:1) (mobile phase B). The combined chromatographic flow rate was 0.65 ml/min, and acetonitrile was infused, post-column, at 0.15 ml/min. Tandem mass spectrometry analysis was performed using an AB SCIEX 6500 triple quadrupole with a Turbo IonSpray source (Foster City, CA) with a Peak Scientific (Scotland, United Kingdom) Table-N_2_ gas generator. Quantitation was achieved by monitoring the native compound transition, 353.3 to 193 m/z (quantitative) and 353.3 to 291 m/z (qualitative), with the corresponding isotopically labelled internal standard transition, 357.3 to 197 m/z. The total cycle time for this method was 11 min.

### Method Validation

Accuracy for this assay was assessed through spike-and-recovery analyses of blank and spiked BAL fluid with known concentrations. For determining accuracy of both the pools and the individual samples, A pool of BAL fluid samples and an individual BAL fluid sample (BAL fluid 1 and BAL fluid 2, respectively, in Table 1) was spiked with three different concentration levels of native 8-isoprostane (200, 500, and 2,000 pg/ml) and compared the spiked concentrations to the initial measurement. Each sample was prepared in triplicate and measured using two different runs spanning 2 days, resulting in a total of 12 samples per spiking level. The concentration from each triplicate sample was then averaged to get the mean concentration of 8-isoprostane for that spiking level. The mean concentration values were used to calulate percent recovery (equation shown in Table1).

**TABLE 1:** Accuracy and spike recovery of 8-isoprostane in BAL fluid (pg/ml).

	Replicate	BAL Fluid 1					BAL Fluid 2				
		Spike concentration	Measured concentration			Recovery^a^ (%)	Spike oncentration	Measured concentration			Recovery^a^ (%)
			Day 1	Day 2	Mean			Day 1	Day 2	Mean	
Initial BAL	1	0	32.2	29.4	35.6	N/A	0	0	0	0.0	N/A
	2		37.2	37.9				0	0		
	3		39.9	36.9				0	0.0		
BAL + spike 1	1	200	230	228	226.8	95.6	200	192	215	203.5	101.8
	2		230	244				207	192		
	3		218	211				212	203		
BAL + spike 2	1	500	494	539	531.8	99.3	500	473	482	492.5	98.5
	2		532	553				505	501		
	3		529	544				476	518		
BAL + spike 3	1	2000	1900	2000	1953	95.9	2000	1950	1900	1910	95.5
	2		1910	1970				1930	1920		
	3		1930	2010				1850	1910		

a Recovery % = [(mean of measured concentration with spike—mean of measured concentration without spike)/spike concentration].

Precision within a run and between runs was determined by using duplicate samples from two BAL fluid pools spiked with native 8-isoprostane at concentrations 200 and 2,000 pg/ml. These results were obtained from five analytical runs over the span of 3 days. The coefficient of variation (CV) was calculated to evaluate both within-run and between-run variation (Table 2).

**TABLE 2:** Precision of quantitation of 8-isoprostane in BAL fluid (pg/ml).

Quality material 1				Quality material 2			
Run	Result 1	Result 2	Mean	Run	Result 1	Result 2	Mean
1	230	230	230	1	1930	1910	1920
2	234	237	235	2	1940	2010	1975
3	228	244	236	3	1970	2000	1985
4	229	232	230	4	1960	2030	1995
5	220	230	225	5	1950	1950	1950
**Quality material 1**				**Quality material 2**		
		**%CV**			**%CV**	
**Within run**		1.95		**Within run**	1.36		
**Between run**		1.92		**Between run**	1.54		

Stability testing was done with two spiked BAL fluid pools of concentrations 500 and 2,000 pg/ml. Six samples from each pool were aliquoted and tested for their initial concentrations. The three test conditions were designed to simulate common sample preparation scenarios, freeze-thaw stability, benchtop stability, and processed sample stability. To test freeze-thaw stability, two samples from each pool were frozen at −70°C and then thawed a room temperature three times each. Testing benchtop stability was done by leaving two samples from each pool out at room temperature for 24 h. To test processed sample stability, samples were left in a collection plate for 24 h in an autosampler set to 4°C, before being reinjected. Results from the initial measurements were then compared to the measurements following the stability testing.

**FIGURE 1:**
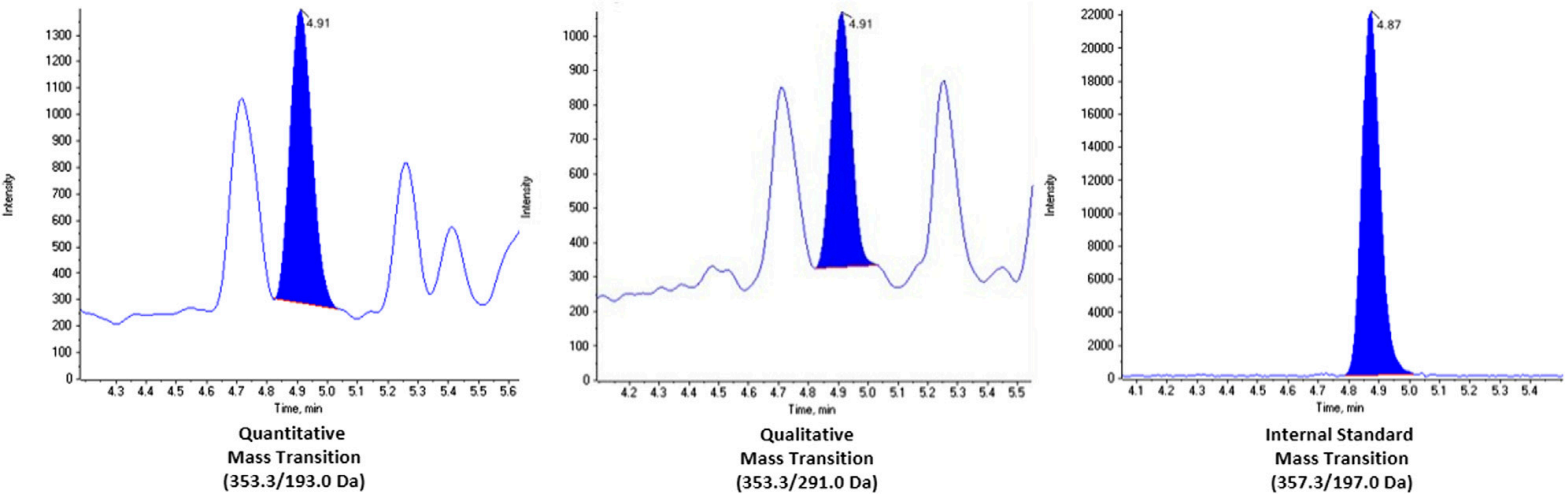
Chromatograms of real BAL fluid sample with concentration 437 pg/ml. The mass transitions for each channel are as follows: quantitative (353.3/193.0 Da), qualitative (353.3/291.0 da), and internal standard (353.3/197.0 Da).

We looked at potential chromatographic interferences in 18 individual BAL fluid samples and none were observed. Representative chromatograms of a real BAL fluid sample, with a calculated concentration of 437 pg/ml, are shown in Figure 1. Additional chromatograms of extracted saline blanks, saline spikes and spiked BAL fluid pools are shown in the Supplemental Figure S1. Multiple ion transitions (quantitative, qualitative, and isotopically labelled internal standard) were monitored to ensure the method was selective to a single compound, 8-isoprostane.

Limit of detection (LOD) was determined by analyzing four BAL fluid pools with known 8-isoprostane concentrations (0–2,000 pg/ml) in triplicate on 5 separate runs spanning 3 days. The standard deviation of each pool was plotted against the mean concentrations, and found the *Y*-intercept, which represents the standard deviation at zero-spike (S_0_). The LOD was defined as 3 times S_0_. (Taylor, 1987)

## Results and Discussion

### Accuracy and Linearity

Accuracy was calculated by comparing the obtained mean concentration with the native 8-isoprostane spiking level, with values that ranged from 95.5–101.8%. The mean recovery was determined to be 97.8% with a standard deviation of 2.5% (Table 1). Our previous assay of 8-isoprostane in urine resulted in recoveries ranging from 92.7 to 106.7% with a mean recovery of 99.7%, indicating that the results from our BAL fluid assay are consistent from analysis of a different physiological matrix. (Holder et al., 2020) We compared the ISTD responses between unextracted calibration samples and extracted samples to evaluate sample recovery and calculated an average recovery of 55% for all extracted samples.

As outlined in our previous assay, the calibration curve was prepared by spiking 10 known standard levels of 8-isoprostane in water, with concentrations ranging from 8.8 to 1,410 pg/ml. (Holder et al., 2020) Each standard level was run in duplicate for each analytical run. Strong linearity was observed with an *R* ^2^ of 0.9999.

### Precision

The within run precision ranged from 1.36–1.95%, and the between run precision ranged from 1.54–1.92% (Table 2). Thus our BAL fluid method was more precise than our previously published urine assay, possibly because BAL fluid is a cleaner matrix. Furthermore, our BAL fluid method had substantially better precision than a typical EIA assay (e.g., Cayman EIA within run: 9.5%, between run: 20.2%). (Cayman Chemical 8-Isopros, 1635)

### Selectivity

Fully resolving 8-isoprostane from all interfering peaks is critical to achieving a reliable and repeatable measurement since this analyte belongs to a class of compounds, F2-isoprostanes, consisting of 64 isomers. Immunoassays are known to suffer from cross-reactivity which could explain the reported poor agreement between LC-MS and EIA for 8-isoprostane, and while GC-MS can be extremely sensitive, extensive sample preparation using harsh derivatizing agents is a necessity, making the GC-MS approach less desirable. (Klawitter et al., 2011; Smith et al., 2011) It is important to note that some published LC-MS/MS methods are not highly selective and measure a sum of F2-isoprostanes and not 8-isoprostane specifically. (Taylor et al., 2008) To evaluate possible interference with 8-isoprostane, we examined the following eicosanoids with similar mass transitions: ent-8-iso-15(S)-PGF_2α_; ent-8-iso-PGF_2α_; 8-iso-PGE_1_; PGE_1_; 8-iso-15(R)-PGF_2α_; PGF_2α_; and 15(R)-PGF_2α_. Our UHPLC-MS/MS method fully resolves the analyte from all interfering peaks and can reliably be used to quantify 8-isoprostane in BAL fluid and other matrixes.

### Stability

Percent differences of the initial measurements and post-test measurements ranged from -0.8–2.1% across all three test conditions and both pools (Table 3). These results indicate that 8-isoprostane is stable under all three conditions tested. Long term stability was not assessed due to the rapid method development timeline dictated by the emergency response.

**TABLE 3:** 8-isoprostane stability in BAL fluid (pg/ml).

	Initial measurement	3 freeze-thaw cycles	Bench-top stability	Processed sample stability
Replicate 1	529	543	540	522
Replicate 2	532	510	539	535
Mean	530.5	526.5	539.5	528.5
% Difference from initial measurement	--	-0.8	1.7	-0.4
	**Initial measurement**	**3 freeze-thaw cycles**	**Bench-top stability**	**Processed sample stability**
Replicate 1	1910	1950	1970	1940
Replicate 2	1930	1920	1950	1940
Mean	1920	1935	1960	1940
% Difference from initial measurement	--	0.8	2.1	1.0

While working in a high throughput laboratory, samples may undergo many freeze-thaw cycles and or be left in an autosampler over the weekend. The results of these tests show that 8-isoprostane levels did not change in samples that underwent the tested conditions, and thus can be used for future analyses. Furthermore, being a stable and robust analyte further supports the use of 8-Isoprostane as a key biomarker of oxidative stress usable in high throughput studies.

### Limit of Detection and Calibration Range

The method detection limit for 8-isoprostane (8.8 pg/ml) was calculated as 3 times S_0_. (Taylor, 1987) We ultimately set the LOD to 17.6 pg/ml for the EVALI samples as we used half the sample volume for analysis due to limited supply. Applications of this method that require an LOQ can use 10 S_0_ (29.3 pg/ml). The calibration range for this method is 8.8 pg/ml to 1,410 pg/ml; our lowest calibrator is lower than our set LOD and a typical deviation is less than 5% of the target concentration. However, Malli et al. measured 8-isoprostane, using EIA, in serum and BAL fluids of patients with either sarcoidosis or idiopathic pulmonary fibrosis (IPF) and found similar concentrations in serum and BAL. They reported median (25–75% interquartile range) concentrations of 8-isoprostane in sarcoidosis patients [serum: 132.8 (92.27–194.9) pg/ml; BAL: 220.6 (133.6–403.3) pg/ml] and IPF patients [serum: 77.25 (52.42–162.5) pg/ml; BAL: 74.87 (62.23–115.1) pg/ml]. (Malli et al., 2013) Bastani and others applied their LC-MS/MS method measuring 8-isoprostane in plasma, urine, full blood, and erythrocytes. (Bastani et al., 2009) To our knowledge there are no other LC-MS methods for measuring 8-isoprostane in BAL fluid, however, our calibration curve is appropriate for reported BAL fluid 8-isoprostane concentrations using EIA. It is difficult to compare the results of LC-MS methods to EIA methods due to the differences in selectivity, so LODs between the two methods cannot be compared. (Klawitter et al., 2011; Smith et al., 2011) Additionally, the volume of BAL fluid obtained from a patient can vary according to technique used to obtain it. Thus, we only report qualitative results for the BAL fluid samples in this study. (De Jesús et al., 2020; Morel Espinosa et al., 2021) Of the 18 samples that were tested using this method, 14 of them had a concentration above the 17.6 pg/ml LOD.

## Conclusion

We have developed and validated a partially automated, selective, and robust UHPLC-MS/MS method for quantifying 8-isoprostane in bronchoalveolar lavage (BAL) fluid. This method is easily adaptable for high-throughput work flow and will be applied to BAL fluid samples collected from EVALI case patients in support of CDC’s 2019 EVALI response. Although EVALI has been strongly linked to inhaled vitamin E acetate from EVPs (Blount et al., 2020), the pathology of how vitamin E acetate causes lung injury remains uncharacterized and may involve OS.

## Data Availability

The original contributions presented in the study are included in the article/Supplementary Material, further inquiries can be directed to the corresponding authors.
