# Supplementary figures and images for: A Novel UHPLC-MS/MS Method for Measuring 8-iso-Prostaglandin F2α in Bronchoalveolar Lavage Fluid
Source: Front Chem. 2021 Aug 12;9:695940. doi: 10.3389/fchem.2021.695940 (PMC8406638; doi:10.3389/fchem.2021.695940)

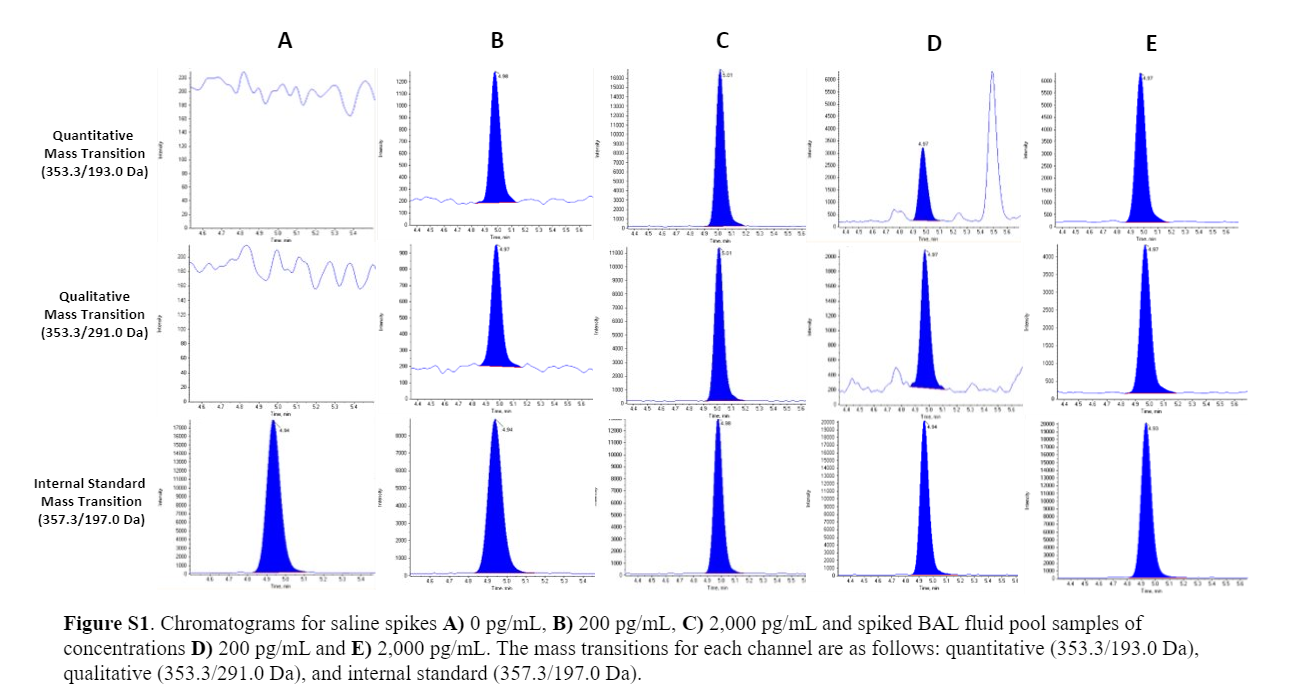

Supplement: Supplementary file 1 [file Image1.TIF]
